# Influence of epicardial adipose tissue inflammation and adipocyte size on postoperative atrial fibrillation in patients after cardiovascular surgery

**DOI:** 10.14814/phy2.15957

**Published:** 2024-03-28

**Authors:** Hiroyuki Natsui, Masaya Watanabe, Takashi Yokota, Satonori Tsuneta, Yoshizuki Fumoto, Haruka Handa, Matsushima Shouji, Jiro Koya, Kotaro Nishino, Daishiro Tatsuta, Takuya Koizumi, Takahide Kadosaka, Motoki Nakao, Taro Koya, Taro Temma, Yoichi M. Ito, Hatanaka C. Kanako, Yutaka Hatanaka, Shingu Yasushige, Satoru Wakasa, Shuhei Miura, Takahiko Masuda, Naritomo Nishioka, Shuichi Naraoka, Kayoko Ochi, Tomoko Kudo, Tsugumine Ishikawa, Toshihisa Anzai

**Affiliations:** ^1^ Department of Cardiovascular Medicine, Faculty of Medicine and Graduate School of Medicine Hokkaido University Sapporo Japan; ^2^ Institute of Health Science Innovation for Medical Care, Hokkaido University Hospital Sapporo Japan; ^3^ Department of Diagnostic and Interventional Radiology Hokkaido University Hospital Sapporo Japan; ^4^ Department of Molecular Biology, Faculty of Medicine and Graduate School of Medicine Hokkaido University Sapporo Japan; ^5^ Department of Cardiovascular Medicine, Faculty of Medicine and Graduate School of Medicine Kyushu University Fukuoka Japan; ^6^ Center for Development of Advanced Diagnostics Hokkaido University Hospital Sapporo Japan; ^7^ Department of Cardiovascular Surgery, Faculty of Medicine and Graduate School of Medicine Hokkaido University Sapporo Japan; ^8^ Department of Cardiovascular Surgery, Teine Keijinkai Hospital Sapporo Japan; ^9^ Department of Clinical Laboratory Medicine Teine Keijinkai Hospital Sapporo Japan

**Keywords:** adipocyte size, epicardial adipose tissue, inflammatory mediators, mitochondrial respiratory capacity, postoperative atrial fibrillation

## Abstract

Epicardial adipose tissue (EAT) is an active endocrine organ that is closely associated with occurrence of atrial fibrillation (AF). However, the role of EAT in the development of postoperative AF (POAF) remains unclear. We aimed to investigate the association between EAT profile and POAF occurrence in patients who underwent cardiovascular surgery. We obtained EAT samples from 53 patients to evaluate gene expression, histological changes, mitochondrial oxidative phosphorylation (OXPHOS) capacity in the EAT, and protein secretion in EAT‐conditioned medium. EAT volume was measured using computed tomography scan. Eighteen patients (34%) experienced POAF within 7 days after surgery. Although no significant difference was observed in EAT profile between patients with and without POAF, logistic regression analysis identified that the mRNA expression levels of tumor necrosis factor‐alpha (TNF‐α) were positively correlated and adipocyte size in the EAT was inversely correlated with onset of POAF, respectively. Mitochondrial OXPHOS capacity in the EAT was not associated with POAF occurrence; however, it showed an inverse correlation with adipocyte size and a positive correlation with adiponectin secretion. In conclusion, changes in the secretory profile and adipocyte morphology of the EAT, which represent qualitative aspects of the adipose tissue, were present before the onset of AF.

## 1 INTRODUCTION

Atrial fibrillation (AF) is the most common arrhythmia, and its incidence is increasing worldwide (Chugh et al., 2014; Schnabel et al., 2015). Postoperative AF (POAF) may prolong hospital stay after open‐heart surgery, with an increased risk of stroke (Greenberg et al., 2017). Advanced age (Seo et al., 2021), atrial structural and electrical remodeling (Kawczynski et al., 2021, 2022; Lotter et al., 2023), obesity, and metabolic syndrome have been associated with increased POAF frequency (Maesen et al., 2012); however, the precise mechanism underlying its occurrence remains unclear.

The epicardial adipose tissue (EAT) is an active endocrine and paracrine organ that lacks fascial boundaries with the myocardium, and its vascular supply comes from the branches of the coronary arteries (Muhib et al., 2013). Previous clinical studies have shown that increased EAT volume was associated with presence of persistent or paroxysmal AF (Al Chekakie et al., 2010; Thanassoulis et al., 2010; Wong et al., 2011) and its recurrence after catheter AF ablation (Chen et al., 2022; Gaeta et al., 2017). In addition, increased EAT volume was reported to have an association with atrial remodeling including increased atrial fibrosis and reduced intra‐atrial conduction velocity in non‐AF patients (Nalliah et al., 2020). Interestingly, human EAT‐conditioned culture medium (in particular, from AF patients) promoted fibrotic changes in the atrial muscle of rats, which may indicate that some profibrotic products may be secreted by the EAT, resulting in atrial fibrosis (Kira et al., 2020). For maintenance of EAT function, mitochondria are thought to be involved in adipocyte differentiation and adipokine secretion (Koh et al., 2007; Wang et al., 2013; Yu & Zhu, 2004). However, none of former studies comprehensively assessed quantity and quality of EAT in AF patients, and thus, the association between POAF and alterations in EAT profile, particularly concerning inflammation and metabolism, remains fully unexplored.

Here we aimed to evaluate quality aspects of EAT (including gene expression and mitochondrial respiratory capacity in the EAT and protein secretion from EAT‐conditioned medium) as well as adipocyte size and volume of the EAT and explore their impact on POAF occurrence in patients who underwent cardiovascular surgery. Understanding how the EAT contributes to the onset of POAF, including alterations of mitochondrial function, may offer novel insights for preventing and treating this common arrhythmia.

## 2 MATERIALS AND METHODS

### 2.1 Study patients

The study population included 58 patients who showed sinus rhythm (SR) at the time of inclusion and were planned to undergo elective cardiac surgery or thoracic aortic surgery with a median sternotomy at either of Hokkaido University Hospital or Teine Keijinkai Hospital from August 2021 to December 2022. Patients were excluded if they met any of the following criteria: (1) Age <20 years; (2) left ventricular ejection fraction <35%; (3) prior cardiac or thoracic surgery; and (4) emergent surgery. Three patients were excluded due to issues with EAT sampling, including two patients with samples inadequate to measure mitochondrial respiratory capacity and one in whom a sample was unobtainable. Two patients were later found to be ineligible after obtaining consent. The remaining 53 patients were enrolled in our study. The ethics committee of each institution approved the study protocol, and the investigation conformed to the principles outlined in the Declaration of Helsinki. Written informed consent was obtained from each patient before surgery. This study was registered in the UMIN Clinical Trials Registry: UMIN000044765.

### 2.2 EAT sample biopsy and preparation

The EAT samples were obtained as previously described (Nakajima et al., 2019). Briefly, EAT was excised from the fat depot on the anterior surface of the myocardium at or below the level of Rindfleisch fold within the pericardium (Nakajima et al., 2019; Parisi et al., 2019, 2020), before or immediately after initiating extracorporeal circulation. The specimens were divided into several sections for subsequent experiments. The section for measuring mitochondrial respiratory capacity was immediately placed in an ice‐cold relaxing solution (BIOPS, in mmol/L: CaK_2_EGTA (ethylene glycol tetraacetic acid) 2.77, EGTA 7.23, taurine 20, MgCl_2_ 6.56, ATP 5.77, phosphocreatine 15, dithiothreitol 0.5, and 4‐morpholineethanesulfonic acid 50, pH 7.1). Other fragments were stored in PAXgene Tissue (Qiagen, Hilden, Germany; Cat. # 765112) or 10% neutral buffered formalin for histological analysis, frozen in liquid nitrogen, stored at −80°C for nucleic acid extraction, and used to generate the conditioned medium.

### 2.3 Evaluation of EAT volume

We assessed EAT volume using computed tomography (CT) images obtained before surgery. The images were captured under electrocardiogram (ECG)‐triggered and 120 kV tube voltage conditions without contrast materials, and measurements were conducted on 5‐mm slice images. The region of interest (ROI) was manually placed by tracing the epicardium using a commercially available software (Figure S1; XTREK view; J‐MAC System, Sapporo, Japan) (Nakajima et al., 2019). The pixels with CT values ranging from −190 Hounsfield Unit to −30 Hounsfield Unit within the ROI were considered as those of adipose tissue (Yoshizumi et al., 1999). The number of pixels was summed up for each slice and multiplied by the slice thickness to estimate the volume of adipose tissue (Oyama et al., 2011). ROIs were set up for the whole heart, peri‐right atrium, and peri‐left atrium, and the EAT volume was calculated in each region.

### 2.4 Twelve‐lead ECG

Twelve‐lead ECG was performed on all participants at the time of admission before surgery. We measured P‐wave indices related to wave amplitude, duration, and dispersion, focusing on atrial remodeling (Li et al., 2021; Rasmussen et al., 2020; Weinsaft et al., 2014).

### 2.5 Echocardiography

All participants underwent 2‐dimensional transthoracic echocardiography within 6 months before surgery. Data on routinely measured conventional echocardiographic parameters were collected from each institute.

### 2.6 Evaluation of POAF

Postoperative AF was defined as AF lasting ≥5 min and detected within 7 days after the surgery. The onset of POAF was confirmed through the continuous monitoring of ECGs and electronic medical charts.

### 2.7 Nucleic acid extraction and analysis

Nucleic acids from EAT frozen samples were extracted using the NucleoSpin RNA kit (Macherey–Nagel, Düren, Germany; Cat. # 740955.50) combined with the NucleoSpin RNA/DNA buffer set (Macherey–Nagel) following the manufacturer's instructions. In brief, EAT frozen samples were homogenized and dissolved in a lysis solution with a strong reducing agent. The lysate was mixed with 70% ethanol to facilitate conditions for binding of nucleic acids to the NucleoSpin RNA Binding Column. Then, the lysate was centrifuged (for 30 s at 11,000 × *g*) and transferred to Nucleospin RNA Binding Column. The column was washed two times using DNA wash buffer‐1 included in the buffer set, and only DNA was eluted. Subsequently, DNase reaction mixture was added to the column and incubated at room temperature for 15 min. The column was washed with wash buffer‐2/3 and RNA was eluted using RNase‐free water. RNA expression related to inflammation, mitochondrial biogenesis, thermogenesis, browning, and whitening was quantified using NanoString technology with a custom array (NanoString Technologies, Seattle, WA). The custom panel included 26 and 3 housekeeping genes (Table S1), and the data were processed using nSolver software (NanoString Technologies). In the nCounter analysis, 47 cases (15 in the POAF group and 32 in the SR group) were measured and analyzed after excluding cases with poor conditions during a preliminary quality check.

### 2.8 Quantification of mitochondrial DNA copy number

DNA solutions extracted from the EAT samples were directly used for polymerase chain reaction (PCR). PCR reactions were conducted in 20‐μL reaction volume using power SYBR green quantitative PCR mix (Applied Biosystems; Cat. # 4367659) in 96‐well optical reaction plates. Pre‐designed commercial primer sets (Human mtDNA Monitoring Primer Set, Takara Bio, Otsu, Japan; Cat. # 7246), representative genes of mitochondrial DNA (mtDNA; *ND1* and *ND5*) and genomic DNA (gDNA; *SLCO2B1* and *SERPINA1*), were used to amplify mtDNA and gDNA. The reactions were performed on a StepOnePlus™ Real‐Time PCR system (Applied Biosystems). The thermal cycling parameters included an enzyme activation step at 95°C for 10 min, followed by 40 cycles of a denaturation step at 95°C for 15 s, and an annealing or extension at 60°C for 60 s. Melt curve analysis was performed after amplification to assess the specificity of the amplified products. The melt curve analysis was conducted using the following steps: denaturation at 95°C for 15 s, followed by a decrease in temperature to 60°C for 1 min, and then an increase to 95°C at 0.3°C for 15 s while continuously monitoring fluorescence readings. PCR efficiency was determined by running serial dilution standard curves and analyzing the slope, and we confirmed that the slope did not change in either reaction. The delta cycle threshold (Ct) methods were used to calculate DNA levels for each mitochondrial gene relative to each nuclear gene using the equations of $2^{\left({\mathrm{Ct}}_{\text{gDNA}}-{\mathrm{Ct}}_{\text{mtDNA}}\right)}$ as previously described (He et al., 2002; Sustarsic et al., 2018). The two mtDNA/gDNA ratios ($2^{\left({\mathrm{Ct}}_{\text{SLCO}2B1}-{\mathrm{Ct}}_{\mathrm{ND}1}\right)}$ and $2^{\left({\mathrm{Ct}}_{\text{SERPINA}1}-{\mathrm{Ct}}_{\mathrm{ND}5}\right)}$) were averaged to obtain the final mtDNA/gDNA ratio according to the manufacturer's instructions.

### 2.9 Mitochondrial respiratory capacity in the EAT

Mitochondrial respiratory capacity in permeabilized EAT was measured on the same day, within 6 h post‐surgery, using a high‐resolution respirometry (Oxygraph‐2 k, Oroboros instruments, Innsbruck, Austria) at 37°C, as previously described (Nakajima et al., 2019). The EAT samples were carefully resected to remove the capillaries and connective tissues. Subsequently, the tissues were placed in 3 mL of MiR05 solution (in mmol/l: sucrose 110, K‐lactobionate 60, EGTA 0.5, 0.1% BSA, MgCl_2_ 3, taurine 20, KH_2_PO_4_ 10, and HEPES 20, pH 7.1). Approximately 50 mg of the sample tissue was placed in a respirometer chamber filled with 2 mL of MiR05.

After permeabilization with 2 μmol/L of digitonin (Fluka; Cat. # 37008), the following respiratory substrates, ADP, and an uncoupler were added to the chamber: (1) 10 mmol/L of glutamate (Sigma‐Aldrich; Cat. # G1626) and 2 mmol/L of malate (Sigma‐Aldrich; Cat. # M1000) (complex I‐linked substrates); (2) 5 mmol/L of ADP (Calbiochem; Cat. # 216201); (3) 0.15 mmol/L of octanoyl‐l‐carnitine (Tocris Bioscience; Cat. # 0605) (a fatty acid); (4) 10 mmol/L of succinate (Sigma‐Aldrich; Cat. # S2378) (a complex II‐linked substrate); (5) 10 μmol/L of cytochrome *c* (Sigma‐Aldrich; Cat. # C7720); and (6) titration of 0.5 μmol/L of carbonylcyanide p‐trifluoromethoxyphenylhydrazone (FCCP; Sigma‐Aldrich; Cat. # C2920) (an uncoupler of oxidative phosphorylation). Cytochrome *c*, which induces an increase in the oxygen (O_2_) consumption rate when the outer mitochondrial membrane is damaged, was added to the chamber to assess the integrity of the outer mitochondrial membrane. We excluded the data in one case because the increase in the O_2_ consumption rate was greater than 10% after adding cytochrome *c*. The O_2_ consumption rate was normalized to the tissue mass and expressed as pmol/s/mg wet weight of EAT. Additionally, it was normalized to the mitochondrial DNA copy number (mtDNA/gDNA), a marker of mitochondrial content, and expressed as pmol/s/mg/mtDNA/gDNA (Kraunsoe et al., 2010; Pedersen et al., 2022).

### 2.10 Preparation of conditioned medium

We prepared conditioned medium to evaluate the secretome of human EAT, as previously described (Kira et al., 2020). Approximately 50 mg of the EAT sample were cut into smaller pieces weighing 10 mg or less. The tissues were washed with phosphate‐buffered saline (PBS) and centrifuged three times for 1 min (400 × *g* at 24°C). Following this, the tissues were cultured overnight in 0.4 mL (10 mg of EAT) of Dulbecco's modified eagle medium F12 (DMEM F12, Gibco, Gland Island, NY) with 5% fetal bovine serum (FBS, Sigma‐Aldrich, St. Louis, MO) and penicillin/streptomycin. After preincubation, the EAT samples were washed three times with PBS and cultured in DMEM F12 without FBS. After 24 h of incubation, the culture supernatant was collected and stored at −80°C.

### 2.11 Secretome in conditioned medium of EAT

The protein levels of adiponectin, interleukin 1 beta (IL‐1β), IL‐6, IL‐8, total plasminogen activator inhibitor 1 (PAI‐1), monocyte chemotactic protein 1 (MCP‐1), resistin, and tumor necrosis factor‐alpha (TNF‐α) in the conditioned medium were measured using Luminex XMAP technology Magpix™ and the human adipokine magnetic bead panel I (Millipore, Billerica, MA; Cat. # HADK1MAG‐61 K), following the manufacturer's protocol. Fluorescence intensity measurements were performed using Luminex100/200™ (R&D Systems Inc., Minneapolis, MS), and analysis was performed using Q‐analyzer software (RayBiotech, Peachtree Corners, GA). IL‐1β and TNF‐α were excluded from the analysis owing to their extremely low levels within the sensitivity range of the assay kit in most cases.

### 2.12 Histology

The EAT samples were fixed with the PAX gene tissue system (Qiagen) or 10% neutral buffered formalin and embedded in paraffin. The sections (4 μm) were stained with hematoxylin and eosin. The adipocyte size was measured using BZ‐X analyzer software (KEYENCE, Osaka, Japan), and the average value from three different sections was used for analysis.

### 2.13 Statistical analysis

Continuous variables are presented as mean ± standard deviation (SD), standard error of the mean, or median with interquartile range, as appropriate. Continuous variables between two groups were compared using unpaired *t*‐test (for normally distributed data) or Mann–Whitney *U* test (for non‐normally distributed data). Chi‐square or Fisher's exact test was used to compare categorical variables. We conducted Pearson's correlation analysis to determine linear relationships between continuous variables. We performed the logistic regression analysis to identify independent risk factors for POAF using variables including age, sex, comorbidities, electrocardiographic or echocardiographic parameters, and EAT volume (which have been reported as potential risk factors for POAF) evaluated using a CT scan. Molecular and morphological parameters, or mitochondrial function in EAT were used as explanatory variables in the logistic regression model. Statistical analyses were performed using the JMP Pro version 17 (SAS Institute Inc., Cary, NC) and GraphPad Prism version 9 (GraphPad Software, San Diego, CA). Statistical significance was set at a value of *p* < 0.05.

## 3 RESULTS

### 3.1 Patient characteristics

Table 1 presents the preoperative characteristics of the total cohort of patients (*n* = 53) who underwent elective cardiovascular surgery. The mean age of the study participants was 68.3 ± 9.7 years, and 31 patients (58%) were men. Within 7 days after surgery, POAF was identified in 18 (34%) patients in the total cohort, and there was no significant difference in the occurrence of POAF between male and female (36% vs. 32%, *p* = 0.781). Most patients had hypertension (72%) or dyslipidemia (68%). Twenty‐six (49%) and 17 (32%) patients had coronary artery disease and diabetes mellitus, respectively. No significant differences in preoperative clinical and anthropometric parameters were observed between the POAF and SR groups (patients who maintained SR even after surgery). In addition, no significant differences were observed in the surgical procedures and procedure times between the two groups (Table S2).

**TABLE 1:** Clinical characteristics of the study patients.

	Total (*n* = 53)	POAF (*n* = 18)	SR (*n* = 35)	*p* Value
Age, year	68.3 ± 9.7	70.8 ± 7.4	67.1 ± 10.6	0.193
Male	31 (58)	11 (61)	20 (57)	0.781
Weight, kg	61.6 ± 12.4	60.0 ± 11.8	62.4 ± 12.8	0.506
BMI, kg/m^2^	23.7 ± 3.9	23.0 ± 3.6	24.1 ± 4.0	0.371
Comorbidities				
Hypertension	38 (72)	15 (83)	23 (66)	0.215
Diabetes	17 (32)	4 (22)	13 (37)	0.358
Dyslipidemia	36 (68)	18 (72)	23 (66)	0.760
Coronary artery disease	26 (49)	9 (50)	17 (48)	0.922
Chronic kidney disease	25 (47)	10 (56)	15 (43)	0.381
Hospitalization for heart failure	8 (15)	4 (22)	4 (11)	0.421
Laboratory data				
BUN, mg/dl	18.7 (13.7–21.9)	20.0 (15.3–22.2)	17.0 (13.7–21.6)	0.322
Creatinine, mg/dl	0.88 (0.71–1.10)	1.01 (0.74–1.15)	0.86 (0.71–1.07)	0.970
Triglyceride, mg/dl	115 (68.5–171.5)	110 (66–174)	115 (77–172)	0.504
Total cholesterol, mg/dl	173 (142–207)	172 (142–195)	173 (142–208)	0.937
HDL cholesterol, mg/dl	52 (45–64)	53 (41–68)	51 (45–61)	0.682
LDL cholesterol, mg/dl	93 (70–119)	90 (53–120)	93 (77–116)	0.625
HbA1c, %	5.8 (5.6–6.6)	5.7 (5.5–6.5)	5.9 (5.6–6.7)	0.414
CRP, mg/dl	0.08 (0.04–0.18)	0.07 (0.05–0.25)	0.08 (0.03–0.14)	0.527
Medication				
β‐blockers	21 (40)	8 (44)	13 (37)	0.607
ACEIs/ARBs	29 (55)	9 (50)	20 (57)	0.621
Statins	34 (64)	10 (56)	24 (69)	0.380
Antidiabetics	17 (32)	4 (22)	13 (37)	0.358
Diuretics	15 (28)	7 (39)	8 (23)	0.220

*Note*: Data are presented as mean ± SD, median (interquartile range) or *n* (%).

Abbreviations: ACEI, angiotensin converting enzyme inhibitor; ARB, angiotensin receptor blocker; BMI, body mass index; BUN, blood urea nitrogen; CRP, c‐reactive protein; HbA1c, hemoglobin A1c; HDL, high‐density lipoprotein; LDL, low‐density lipoprotein; POAF, postoperative atrial fibrillation; SR, sinus rhythm.

Table 2 presents the results of the detailed P‐wave analyses in the 12‐lead ECG and echocardiographic parameters performed before surgery. The maximum and minimum P‐wave durations in lead II, P‐wave amplitude in lead V1, P‐wave terminal force in lead V1 (PTFV1), and P‐wave area in lead V1 were greater in the POAF group than in the SR group. No differences were observed in the left ventricular end‐diastolic diameter, left ventricular end‐systolic diameter and left ventricular ejection fraction between the two groups, although the left atrial diameter was significantly larger (45.1 ± 6.5 mm vs. 39.6 ± 6.5 mm; *p* = 0.006) and left atrial volume index (LAVI) tended to be larger in the POAF group (58.0 vs. 38.2 mL/m^2^; *p* = 0.071). The preoperative EAT volume evaluated using CT was comparable between the POAF and SR groups (Table 3). In addition, the EAT volume did not correlate with electrocardiographic or echocardiographic parameters (data not shown).

**TABLE 2:** Twelve‐lead ECG and echocardiographic characteristics.

	Total (*n* = 53)	POAF (*n* = 18)	SR (*n* = 35)	*p* Value
ECG				
PR interval, msec	174 (159–192)	175 (163–192)	172 (156–194)	0.607
Minimum P duration, msec	102 (92–117)	110 (99–120)	101 (90–110)	0.015
Maximal P duration, msec	130 (120–140)	138 (120–146)	127 (117–136)	0.019
P‐wave dispersion, msec	24 (19–36)	24.5 (20–35.5)	22 (16–36)	0.730
P duration (V1), msec	53 (44–66)	52 (46–71)	53 (43–65)	0.618
P amplitude (V1), 10^−2^ mV	5.4 (3.6–6.4)	6.3 (5.1–8.0)	4.3 (3.2–5.8)	0.013
P′ duration (V1), msec	64 (55–75)	73 (61–83)	59 (51–71)	0.090
P′ amplitude (V1), 10^−2^ mV	5.0 (3.8–7.2)	6.3 (4.8–7.9)	4.7 (3.2–7.2)	0.213
PTFV1, msec*mV	3.5 (2.0–5.3)	4.3 (3.5–6.2)	2.9 (1.9–4.2)	0.044
P wave area, msec*mV	6.5 (4.7–8.4)	7.5 (6.5–13.0)	5.8 (4.3–7.5)	0.007
QRS interval, msec	98 (88–116)	94 (87–117)	103 (89–116)	0.397
UCG				
IVST, mm	10.7 ± 2.0	10.7 ± 1.9	10.7 ± 2.0	0.956
LVPWT, mm	9.6 ± 1.8	9.8 ± 2.0	9.5 ± 1.7	0.612
LVDd, mm	49.8 ± 7.9	50.0 ± 9.1	49.6 ± 7.4	0.863
LVDs, mm	35.0 ± 7.9	35.1 ± 9.3	34.9 ± 7.2	0.962
LVEF, %	57 (47–65)	61 (47–66)	55 (47–64)	0.626
E, cm/s	66.5 (56.5–95.2)	74.1 (55.0–112.0)	66.1 (56.6–88.5)	0.181
A, cm/s	81.2 (64.2–93.9)	82.3 (67.1–93.0)	80.3 (60.7–94.9)	0.307
E/A	0.8 (0.7–1.2)	0.95 (0.65–1.29)	0.80 (0.70–1.20)	0.936
E/e’	11.0 (8.2–13.4)	12.5 (9.3–18.6)	9.2 (7.6–12.5)	0.046
LAD, mm	41.5 ± 7.0	45.1 ± 6.5	39.6 ± 6.5	0.006
LAVI, mL/m^2^	46.4 (33.5–62.6)	58.0 (45.9–69.4)	38.2 (28.2–57.3)	0.071

*Note*: Data are presented as mean ± SD or median (interquartile range).

Abbreviations: ECG, electrocardiogram; IVST, interventricular septum thickness; LAD, left atrial dimeter; LAVI, left atrial volume index; LVDd, left ventricular end‐diastolic diameter; LVDs, left ventricular end‐systolic diameter; LVEF, left ventricular ejection fraction; LVPWT, left ventricular post wall thickness; POAF, postoperative atrial fibrillation; PTFV1, P wave terminal force in lead V1; SR, sinus rhythm; UCG, ultrasound cardiogram.

**TABLE 3:** Preoperative EAT volume evaluated by CT imaging.

	Total (*n* = 53)	POAF (*n* = 18)	SR (*n* = 35)	*p* Value
EAT volume				
Total EAT, cm^3^	171 (131–253)	150 (119–193)	183 (132–284)	0.144
RA EAT, cm^3^	15.7 (11.6–20.0)	15.0 (12.2–16.6)	16.0 (11.2–23.1)	0.364
LA EAT, cm^3^	20.0 (11.2–30.1)	18.1 (13.1–22.5)	21.1 (11.1–33.8)	0.257

*Note*: Data are expressed as median (interquartile range). EAT volume was analyzed separately for the whole heart (Total EAT), around RA (RA EAT) and around LA (LA EAT).

Abbreviations: EAT, epicardial adipose tissue; LA, left atrium; POAF, postoperative atrial fibrillation; RA, right atrium; SR, sinus rhythm.

### 3.2 Mitochondrial respiratory capacity in EAT

**FIGURE 1:**
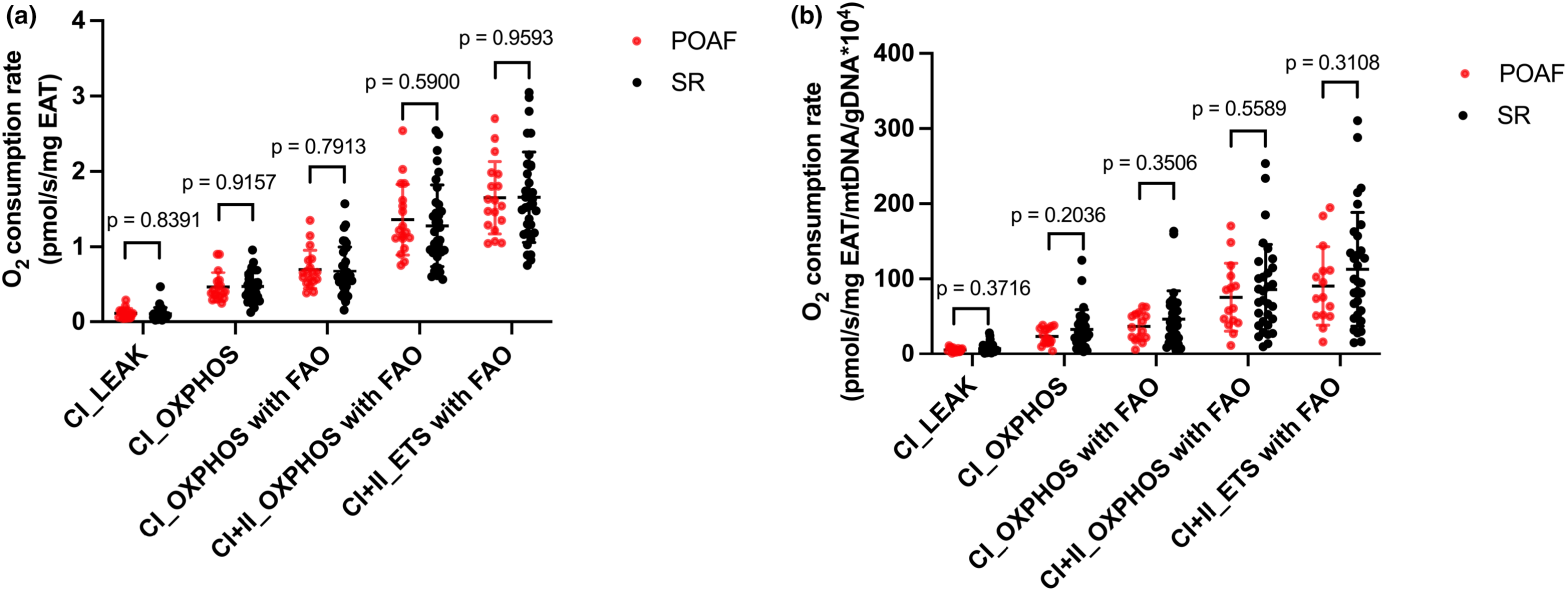
Mitochondrial respiratory capacity in epicardial adipose tissue (EAT). (a) O_2_ consumption rate (pmol/s/mg EAT) at each respiratory state using non‐fatty and fatty acid substrates. (b) O_2_ consumption rate normalized to mitochondrial DNA copy number (pmol/s/mg EAT/mtDNA/gDNA × 10^4^). The respiratory states in (a) and (b) were not significantly different between the POAF (*n* = 18) and SR (*n* = 35) groups. Bar: mean ± SD. POAF, postoperative atrial fibrillation; SR, sinus rhythm; CI, complex I‐linked substrates; CI + II, complex I + II‐linked substrates; ETS, maximal electron transfer system capacity; FAO, fatty acid oxidation; LEAK, leak‐state respiration (i.e., non‐ADP stimulated respiration); OXPHOS, oxidative phosphorylation capacity (i.e., ADP‐stimulated respiration).

Figure 1a shows the data on EAT mitochondrial respiratory capacity. No significant difference was observed in mitochondrial respiration normalized to the wet weight of EAT at each respiratory state between the POAF and SR groups. The mitochondrial respiratory capacity, which was normalized to the mtDNA copy number, did not differ between the POAF and SR groups (Figure 1b).

### 3.3 Adipocyte size of EAT

**FIGURE 2:**
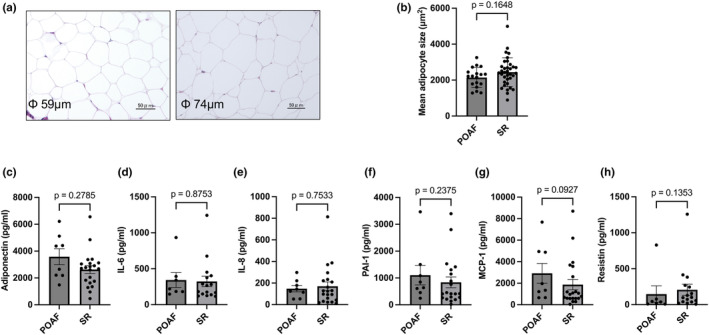
Histological study, and the protein content in the EAT secretome. (a) Representative histological images of the EAT stained with hematoxylin and eosin. The right image shows the EAT with small adipocytes (mean short diameter was 59 μm), and the left image shows the EAT with large adipocytes (mean short diameter was 74 μm). (b) Bar graph showing mean adipocyte size (μm^2^) in the POAF and SR groups. (c–h) Protein content of adiponectin, IL‐6, IL‐8, PAI‐1, MCP‐1, and resistin in the EAT secretome. The number of adiponectin, IL‐8, and MCP‐1 samples was 8 and 21 in the POAF and SR groups, respectively. For other protein expression levels, except for a few patients that could not be measured successfully, the number of samples was 7 and 16 for IL‐6 and resistin and 8 and 20 for PAI‐1. Bar: mean ± SD. POAF, postoperative atrial fibrillation; SR, sinus rhythm; IL‐6, interleukin‐6; IL‐8, interleukin‐8; PAI‐1, plasminogen activator inhibitor‐1; MCP‐1, monocyte chemoattractant protein‐1. Statistical comparisons were performed using an unpaired *t*‐test.

Figure 2a,b show the histological assessment of the EAT. No significant difference was observed in the mean adipocyte size between the POAF and SR groups (mean ± SD: 2145 ± 559 μm^2^ vs. 2442 ± 798 μm^2^, Figure 2b).

### 3.4 Protein content in conditioned medium from EAT

We measured protein levels in the conditioned medium from 36 subjects; levels were not measured in the remainder due to limited EAT samples. We ultimately analyzed proteins in the conditioned medium from 8 POAF and 21 SR patients, excluding some subjects with exceedingly low or undetectable protein concentrations in the conditioned medium. Representative adipokines and pro‐inflammatory cytokines in the conditioned medium, such as adiponectin, IL‐6, IL‐8, PAI‐1, MCP‐1, and resistin, were measured to evaluate the secretome profile of the EAT (Figures 2c–h). No significant differences were observed in the secreted protein levels originating from human EAT in the conditioned medium between the two groups. The protein content of TNF‐α in conditioned medium was below the lowest level detectable in most cases. However, the protein content of adiponectin in the conditioned medium was positively correlated with mitochondrial oxidative phosphorylation (OXPHOS) capacities normalized to the mg wet weight of EAT (OXPHOS with complex I‐linked substrates, *r* = 0.387, *p* = 0.038; OXPHOS with complex I‐linked substrates and fatty acids, *r* = 0.494, *p* = 0.006; OXPHOS with complex I + II‐linked substrates and fatty acids, *r* = 0.403, *p* = 0.03, data not shown).

### 3.5 Gene expression of EAT

Table 4 shows mRNA expression level in the EAT. Gene expression levels were expressed as normalized counts, which were calculated with a two‐step normalization process using nSolver software (NanoString Technologies). The normalization was performed with a within‐sample normalization using the observed values of positive control probes and a normalization across samples using housekeeping genes (*GAPDH*, *PGK1*, *and PPIA*) (Aguado et al., 2021). The gene expression of mitochondrial transcription factor A (*TFAM*), which is related to mitochondrial biogenesis, in the EAT tended to be lower in the POAF group than that in the SR group (*p* = 0.054). However, no significant differences were observed in the mRNA expression levels of other measured mRNA targets between the POAF and SR groups.

**TABLE 4:** mRNA expression in the EAT.

	POAF (*n* = 15)	SR (*n* = 32)	*p* Value
ADIPOQ	17,086 (12,766–27,004)	17,406 (13,558–24,330)	0.991
ADIPOR1	120.1 (104.0–146.9)	112.1 (98.3–123.6)	0.114
ADIPOR2	1215 (995–1504)	1165 (983–1406)	0.627
CCL2	1102 (716–3344)	1564 (956–3566)	0.303
CIDEA	5226 (3756–9221)	4782 (3814–6979)	0.866
GDF15	30.91 (23.26–79.28)	33.53 (26.16–55.20)	0.761
HOXC9	13.11(9.57–30.22)	16.45 (10.60–25.63)	0.901
IL10	73.89 (45.56–98.14)	64.60 (42.25–91.58)	0.778
IL1b	38.6 (23.86–51.57)	53.42 (35.65–90.41)	0.103
IL6	121.7 (71.6–261.0)	142.08 (78.94–1248.19)	0.553
ITLN1	24,475 (7999–75,131)	68,053 (12,863–164,832)	0.252
LEP	2149 (1623–3519)	2848 (2108–4245)	0.108
NFE2L2	2278 (2035–2499)	2405 (2097–2896)	0.130
NRF1	173.2 (145.8–197.4)	169.1 (155.7–196.9)	0.955
PPARGC1A	80.66 (70.84–146.84)	89.08 (62.55–105.66)	0.371
PRKAA1	1060 (993–1088)	1032 (972–1146)	0.991
RETN	17.70 (12.25–39.44)	14.54 (12.22–20.84)	0.357
SIRT1	545.5 (501.0–576.2)	571.1 (523.8–623.4)	0.136
TBX1	162.7 (65.6–369.2)	106.6 (53.2–245.5)	0.225
TFAM	582.5 (521.9–683.5)	547.4 (488.5–587.4)	0.059
TNF‐α	58.25 (34.87–190.5)	60.26 (34.52–83.17)	0.422
TNFRSF9	33.06 (24.12–42.38)	27.93 (20.79–39.72)	0.491
UCP1	60.15 (43.29–100.85)	47.87 (24.46–84.65)	0.155

*Note*: Data are expressed as median (interquartile range) with the unit of normalized count. The details regarding the name of each gene are described in Table S1.

Abbreviations: POAF, postoperative atrial fibrillation; SR, sinus rhythm.

### 3.6 Risk factors of POAF

**FIGURE 3:**
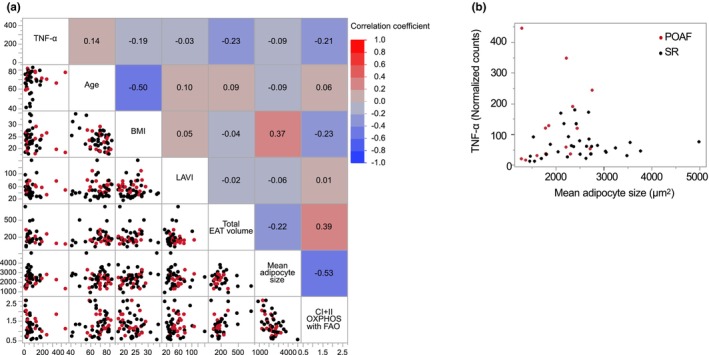
Correlation matrix and heatmap of explanatory variables. (a) Correlation matrix and heatmap of selected explanatory variables that are predictors of POAF in logistic regression analysis. Mitochondrial respiratory capacity in the EAT (CI + II_OXPHOS with FAO) had a negative correlation with mean adipocyte size. (b) The relationship between mRNA expression of TNF‐α and mean adipocyte size. The POAF group is represented by red dots and the SR group by black dots. POAF, postoperative atrial fibrillation; SR, sinus rhythm; TNF‐α, tumor necrosis factor‐alpha; BMI, body mass index; LAVI, left atrial volume index; CI + II, complex I + II‐linked substrates; OXPHOS, oxidative phosphorylation capacity; FAO, fatty acid oxidation.

We performed a logistic regression analysis to determine the independent predictors of POAF after cardiovascular surgery. The best subset selection method (Zhang, 2016) was used to select one or two explanatory variables. The mean adipocyte size and mRNA expression of TNF‐α in the EAT were the best‐fit models to independently predict POAF onset (Table 5). No significant correlation was observed between the mean adipocyte size and mRNA expression of TNF‐α in the EAT of the total cohort (Figure 3a), and this suggested that there was no strong multicollinearity in this analysis. In the scatter plots of TNF‐α and mean adipocyte size, none of the patients exhibited high values of both TNF‐α and adipocyte size, and none of the subjects who experienced POAF had a mean adipocyte size >3000 μm^2^ (Figure 3b).

**TABLE 5:** Logistic analysis for prediction of POAF.

Variables	ORs	95% CI	*p* Value
TNF‐α	1.012	1.001–1.024	0.040
Mean adipocyte size	0.998	0.997–0.999	0.027

Abbreviations: CI, confidence interval; ORs, odds ratios; POAF, postoperative atrial fibrillation; TNF‐α, tumor necrosis factor‐alpha.

### 3.7 Association between mean adipocyte size and mitochondrial respiratory capacity in EAT

**FIGURE 4:**
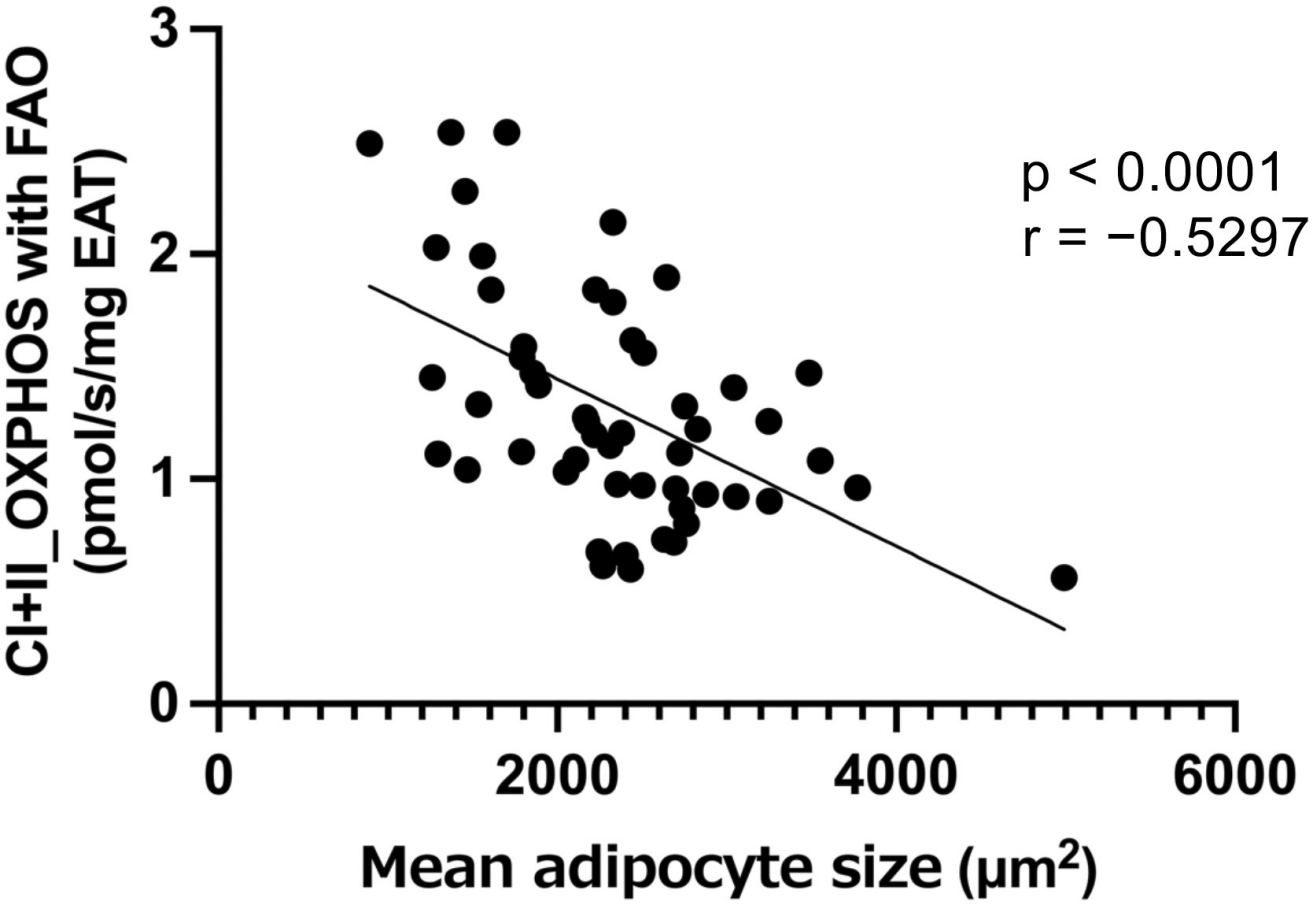
The association between mitochondrial respiratory capacity in the EAT and mean adipocyte size. Mean adipocyte size was inversely correlated with mitochondrial oxidative phosphorylation (OXPHOS) capacity with complex I + II‐linked substrates and fatty acids.

We examined the association between adipocyte size and mitochondrial respiration in the EAT in the total cohort. An inverse correlation was observed between the mean adipocyte size and the mitochondrial OXPHOS capacity with complex I + II‐linked substrates and fatty acids (i.e., maximal OXPHOS capacity) when normalized to the wet weight of EAT (Figure 4). Similarly, the mean adipocyte size was inversely correlated with other mitochondrial respiratory capacities normalized to the wet weight of EAT (LEAK respiration [non‐ADP stimulated respiration] with complex I‐linked substrates, *r* = −0.41, *p* = 0.023; OXPHOS with complex I‐linked substrates, *r* = −0.501, *p* < 0.001; OXPHOS with complex I‐linked substrates and fatty acids, *r* = −0.52, *p* < 0.001, maximal electron transfer system capacity with complex I‐linked substrates and fatty acids, *r* = −0.50, *p* < 0.001). These results did not change after the O_2_ consumption rate of the EAT mitochondria was normalized to the mtDNA copy number (data not shown).

## 4 DISCUSSION

In this study, we explored the relationship between EAT and POAF from a multifaceted perspective by examining EAT volume, gene expression, secretory products, adipocyte morphology, and mitochondrial respiratory capacity in patients who underwent cardiac or thoracic aortic surgery. Our major findings were as follows: (1) mitochondrial respiratory capacity in the EAT was not associated with POAF occurrence; (2) EAT volume measured using CT was not associated with POAF occurrence; and (3) the mean adipocyte size and mRNA expression of TNF‐α in the EAT were the best‐fit explanatory variables predicting POAF occurrence.

### 4.1 Association between EAT volume and occurrence of POAF

The EAT accumulation, as well as obesity and weight gain, is associated with AF progression (Al Chekakie et al., 2010; Tedrow et al., 2010; Thanassoulis et al., 2010; Wong et al., 2011) and atrial structural and electrical remodeling before AF development (Friedman et al., 2014; Nalliah et al., 2020). However, the predictive value of EAT accumulation for POAF development remains controversial (Drossos et al., 2014; Gunturk et al., 2020; Opolski et al., 2015; van der Heijden et al., 2022; Wang et al., 2019). In this study, we could not confirm the relationship between EAT volume and POAF development. Such inconsistent results may be attributable to differences in patients' characteristics. For example, many studies that demonstrating a positive correlation between EAT volume and the development of POAF primarily involved Western individuals with a higher BMI of approximately 30 kg/m^2^. This contrasts with the characteristics of patients in our study and that of Wang Q et al. (2019), both of which focused on patients with a lower BMI of approximately 23 kg/m^2^. On the contrary, increased expression of inflammatory genes may be related to the development of POAF, regardless of BMI. Our results as well as previous studies suggest that the POAF occurrence may depend on qualitative rather than quantitative aspects of the EAT (Petraglia et al., 2022; Wang et al., 2019).

### 4.2 Predictors of POAF occurrence

Logistic regression analysis showed that TNF‐α mRNA expression and adipocyte size of the EAT were independent predictors of POAF occurrence in non‐obese patients who underwent cardiovascular surgery. Previous studies have shown that elevated gene expression of activin A and protein levels of inflammatory proteins in the EAT are associated with the onset of POAF (Cherian et al., 2012; Petraglia et al., 2022; Wang et al., 2019), which is in line with our findings. On the other hand, while prior studies assessed EAT volume in relation to POAF, our study is the first to investigate the association between the EAT adipocyte size and the development of POAF. Although increased adipocyte size is generally associated with detrimental condition including inflammation (Song & Kuang, 2019), recent studies using peri‐coronary EAT and EAT distributed around the heart have suggested that adipocytes may become smaller in relation to the increased release of pro‐inflammatory cytokines in AF patients (Ishii et al., 2021; Mocanu et al., 2020). Interestingly, Mancio et al. (2022) have suggested that impaired EAT adipocyte differentiation and lipid droplet formation are associated with POAF. Although the mechanistic insight into the relationship between adipocyte size and AF largely remains undiscovered, prior studies examining the EAT profile in coronary artery stenosis may help explain the mechanism involved. They demonstrated that inflammatory mediators released from coronary plaques into the surrounding adipose tissue blocked the differentiation of pre‐adipocytes into mature adipocytes and triggered their proliferation resulting in smaller adipocytes (Antonopoulos et al., 2017). Therefore, it might be possible that chronic inflammation in the EAT impaired adipocyte differentiation, which results in changes in adipocyte size. Further studies are necessary to investigate underlying mechanisms of association of alterations in these EAT profiles with POAF occurrence.

### 4.3 Mitochondrial respiratory capacity of EAT

To the best of our knowledge, this study is the first to explore the relationship between the EAT mitochondrial respiratory capacity and POAF development. Unexpectedly, the mitochondrial respiratory capacity of the EAT was not directly associated with POAF development. However, the EAT mitochondrial respiratory capacity normalized to the wet weight of EAT showed an inverse correlation with adipocyte size and a positive correlation with adiponectin secretion in the EAT. These correlations did not change even if mitochondrial respiratory capacity was normalized to the mtDNA copy number, indicating that intrinsic mitochondrial respiration as well as mass‐specific mitochondrial respiration in the EAT were associated with decreased adipocyte size and increased adiponectin secretion in the EAT. In consistent with our findings, a previous study reported a negative correlation between adipocyte size and mitochondrial respiratory capacity in subcutaneous adipose tissue but not in visceral adipose tissue (Honecker et al., 2021). These findings may suggest that the EAT mitochondrial respiratory capacity plays a role in characterizing the EAT profile, and mitochondrial respiratory capacity might influence adipocyte morphology or lipid droplet formation, and vice versa under certain conditions. In addition, changes in the secretory profile and adipocyte morphology of the EAT were observed before the onset of POAF. A recent study indicated increased gene expression of inflammatory cytokines in large adipocytes, which was accompanied by decreased gene expression related to oxidative phosphorylation (Honecker et al., 2022). However, the exact meaning of inverse correlation between mitochondrial respiratory capacity and adipocyte size in the EAT in our study remains unknown.

### 4.4 Clinical implications

Our study demonstrated that changes in the secretory profile and adipocyte morphology of the EAT were present before the onset of AF and that the remodeling of qualitative aspects of the EAT may increase the risk of POAF. Furthermore, our results suggested that an increase in EAT volume might not coincide with a deterioration of its qualitative aspects. Understanding these mechanisms may reveal therapeutic interventions to address qualitative changes in the EAT (Willar et al., 2023) and noninvasive approaches to assess qualitative changes of the EAT (Ishii et al., 2021; Mancio et al., 2022) in terms of reducing the risk of AF.

### 4.5 Study limitations

Our study had some limitations. First, the relatively lower incidence of POAF, which had been reported to be 20% to 40% (Bessissow et al., 2015). This may be owing to the inclusion of patients who underwent cardiac and thoracic aortic open‐heart surgery, as the type of surgery can influence the occurrence rate of POAF (Maesen et al., 2012). Second, we could not identify the causal relationship between the POAF occurrence and mRNA expression level of TNF‐α or adipocyte size of the EAT. Third, the assessment of gene and protein expression was focused on a select group of cytokines and adipokines associated with inflammation and fibrosis. Examining a broader range of inflammatory mediators could have uncovered more significant distinctions in the EAT between the POAF and SR groups. Fourth, the evaluation of EAT secretion was limited to a subset of subjects and may have been inaccurately assessed. Considering the small sample size of this study, the statistical power for detecting significant differences may have been insufficient. Finally, due to the non‐obese cohort, this study might not have fully captured relevant contributions of adipose tissue metabolism to POAF. Future studies including obese individuals with more dysfunctional adipose tissue are desired.

## 5 CONCLUSION

Our study observed no direct association between EAT mitochondrial respiratory capacity and POAF occurrence. However, our data demonstrated that changes in the secretory profile and adipocyte morphology of the EAT, which represent qualitative aspects of the adipose tissue, were present before the onset of AF.

## AUTHOR CONTRIBUTIONS

H.N., M.W., and T.Y. conceived and designed the study; H.N., T.Y., Y.F., H.H., Y.S., S.W., S.M., T.M., N.N., S.N., K.O., T.K. and T.I. performed the experiments; H.N., M.W., T.Y., and Y.I. analyzed the data; H.N., M.W., T.Y., S.M., K.H., Y.H., and Y.I. interpreted the results of the experiments; H.N. prepared the figures; H.N. drafted the manuscript; all authors edited and revised the manuscript and approved the final version of the manuscript for publication.

## FUNDING INFORMATION

This work was partly supported by the Japan Society for the Promotion of Science Grant in Aid for Scientific Research (KAKENHI) Grants 22K08197 (to M. Watanabe), 18K15874 (to T. Temma), and 22K20646 (to T. Kadosaka).

## CONFLICT OF INTEREST STATEMENT

No conflicts of interest, financial or otherwise, are declared by the authors.

## ETHICS STATEMENT

The ethics committee of each institution approved the study protocol (020‐0097) and the investigation conformed to the principles outlined in the Declaration of Helsinki. Written informed consent was obtained from each patient before surgery.

## Supporting information


Appendix S1.

## Data Availability

Data will be made available upon reasonable request.
