# Correction to Influence of epicardial adipose tissue inflammation and adipocyte size on postoperative atrial fibrillation in patients after cardiovascular surgery

**DOI:** 10.14814/phy2.16030

**Published:** 2024-04-26

Natsui, H. , Watanabe, M. , Yokota, T. , Tsuneta, S. , Fumoto, Y. , Handa, H. , Shouji, M. , Koya, J. , Nishino, K. , Tatsuta, D. , Koizumi, T. , Kadosaka, T. , Nakao, M. , Koya, T. , Temma, T. , Ito, Y. M. , Kanako, H. C. , Hatanaka, Y. , Yasushige, S. … Anzai, T. (2024). Influence of epicardial adipose tissue inflammation and adipocyte size on postoperative atrial fibrillation in patients after cardiovascular surgery. Physiological Reports, 12, e15957.38546216 10.14814/phy2.15957PMC10976808

In the “FUNDING INFORMATION” section, the text “This work was partly supported by the Japan Society for the Promotion of Science Grant in Aid for Scientific Research (KAKENHI) Grants 22K08197 (to M. Watanabe), 18K15874 (to T. Temma), and 22K20646 (to T. Kadosaka).” was incorrect. This should have read: “This work was partly supported by the Japan Society for the Promotion of Science Grant in Aid for Scientific Research (KAKENHI) Grants 22K08197 (to M. Watanabe), 18K15874 (to T. Temma), 22K20646 (to T. Kadosaka), and 23K15149 (to T. Koya).”

Authors first name and last name is corrected from Matsushima Shouji is changed to Shouji Matsushima;  Hatanaka C Kanako is changed to Kanako C Hatanaka and Shingu Yasushige is changed to Yasushige Shingu. The author names have been updated in the online version.

We apologize for this error.

