# Supplementary material for: Influence of epicardial adipose tissue inflammation and adipocyte size on postoperative atrial fibrillation in patients after cardiovascular surgery
Source: Physiol Rep. 2024 Mar 28;12(6):e15957. doi: 10.14814/phy2.15957 (PMC10976808; doi:10.14814/phy2.15957)
Supplement: Supplementary file 1 — Appendix S1. [file PHY2-12-e15957-s001.docx]

**Supplementary material**

***Original research article***

**Influence of epicardial adipose tissue inflammation and adipocyte size on post-operative atrial fibrillation in patients after cardiovascular surgery**

**Supplementary tables**

**Supplementary Table S1. NanoString Genes**

| **Gene symbol** | **Official gene name** | **Designation** |
| --- | --- | --- |
| ADIPOQ | adiponectin, C1Q and collagen domain containing | Endogenous |
| ADIPOR1 | adiponectin receptor 1 | Endogenous |
| ADIPOR2 | adiponectin receptor 2 | Endogenous |
| CCL2 | C-C motif chemokine ligand 2 | Endogenous |
| CIDEA | cell death-inducing DFFA-like effector a | Endogenous |
| GDF15 | growth differentiation factor 15 | Endogenous |
| HOXC9 | homeobox C9 | Endogenous |
| IL10 | interleukin 10 | Endogenous |
| IL1b | interleukin 1 beta | Endogenous |
| IL6 | interleukin 6 | Endogenous |
| ITLN1 | intelectin 1 | Endogenous |
| LEP | leptin | Endogenous |
| NFE2L2 | nuclear factor (erythroid-derived 2)-like 2 | Endogenous |
| NRF1 | nuclear respiratory factor 1 | Endogenous |
| PPARGC1A | PPARG coactivator 1 alpha | Endogenous |
| PRKAA1 | protein kinase AMP-activated catalytic subunit alpha 1 | Endogenous |
| RETN | resistin | Endogenous |
| SIRT1 | sirtuin 1 | Endogenous |
| TBX1 | T-box transcription factor 1 | Endogenous |
| TFAM | transcription factor A, mitochondrial | Endogenous |
| TNF-α | tumor necrosis factor alpha | Endogenous |
| TNFRSF9 | TNF receptor superfamily member9 | Endogenous |
| UCP1 | uncoupling protein 1 | Endogenous |
| GAPDH | glyceraldehyde-3-phosphate dehydrogenase | Housekeeping |
| PGK1 | phosphoglycerate kinase 1 | Housekeeping |
| PPIA | peptidylprolyl isomerase A | Housekeeping |
| NEG_A | Negative control A | Negative |
| NEG_B | Negative control B | Negative |
| NEG_C | Negative control C | Negative |
| NEG_D | Negative control D | Negative |
| NEG_E | Negative control E | Negative |
| NEG_F | Negative control F | Negative |
| POS_A | Positive control A | Positive |
| POS_B | Positive control B | Positive |
| POS_C | Positive control C | Positive |
| POS_D | Positive control D | Positive |
| POS_E | Positive control E | Positive |
| POS_F | Positive control F | Positive |

**Supplementary Table S2. Surgical procedural characteristics**

|  | **Total**  (n = 53) | **POAF**  (n = 18) | **SR**  (n = 35) | **p value** |
| --- | --- | --- | --- | --- |
| **Surgical procedure** |  |  |  |  |
| CABG | 16 (30) | 6 (33) | 10 (29) | 0.759 |
| AVR | 11 (20) | 3 (17) | 8 (23) | 0.730 |
| MVR | 12 (23) | 5 (28) | 7 (20) | 0.4105 |
| Aorta replacement | 1 (2) | 0 (0) | 1 (3) | >0.999 |
| CABG + AVR | 3 (5) | 1 (6) | 2 (6) | >0.999 |
| CABG + Aorta replacement | 1 (2) | 0 (0) | 1 (3) | >0.999 |
| AVR + MVR | 3 (5) | 2 (11) | 1 (3) | 0.263 |
| AVR + Aorta replacement | 5 (9) | 1 (6) | 4 (11) | 0.651 |

Data are expressed as n (%). POAF, postoperative atrial fibrillation; SR, sinus rhythm; CABG, coronary artery bypass graft; AVR, aortic valve replacement; MVR, mitral valve replacement/repair.

**Supplementary figure**

**Supplementary Figure S1**

**
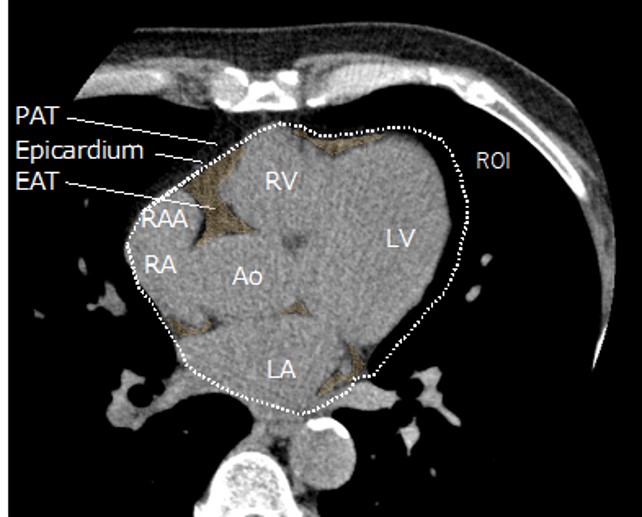
**

**Distribution and quantification of EAT.**

EAT (yellow) located within the epicardium. The region of interest (ROI) was manually placed and the number of pixels within the ROI was calculated in each slice. The number of pixels in each slice (from pulmonary artery bifurcation to the apex) was summed for each slice and multiplied by the slice thickness (5mm).

Ao, aortic root; EAT, epicardial adipose tissue; LA, left atrium; LV, left ventricle; PAT, pericardial adipose tissue; RA, right atrium; RAA, right atrial appendage; ROI, region of interest; RV, right ventricle.
